# Narrative Coherence and Relational Agency: Unraveling Transitions Into and Out of Alberta Correctional Facilities for People Living With HIV

**DOI:** 10.1177/10497323241278537

**Published:** 2024-11-05

**Authors:** Morgan Wadams, Jana Grekul, Sean Lessard, Anthony de Padua, Vera Caine

**Affiliations:** 1 Faculty of Nursing, 3151 MacEwan University, Edmonton, AB, Canada; 2 Faculty of Arts, 3158 University of Alberta, Edmonton, AB, Canada; 3 Faculty of Education, 3158 University of Alberta, Edmonton, AB, Canada; 4 College of Nursing, 7235 University of Saskatchewan, Prince Albert, SK, Canada; 5 School of Nursing, 8205 University of Victoria, Victoria, BC, Canada

**Keywords:** correctional facilities;, HIV;, narrative inquiry;, relational agency;, transitions;, nursing

## Abstract

Incarcerated populations in Canada face significant health and social challenges during transitions into and out of correctional facilities. These transitions around facilities pose disproportionate barriers to care for people living with HIV. Further research is crucial to comprehend these challenges and reimagine care concepts for people who experience structural marginalization. In this article, experiences of transitions into and out of Alberta correctional facilities for people living with HIV are explored using narrative inquiry. Conducted in a Western Canadian city from 2021 to 2022, the inquiry revolved around two men living with HIV and a history of incarceration. Through co-creating field texts and narrative accounts, their unique experiences of transitions were explored through a collaborative process of analysis. Narrative threads from Bruce and Kyle showcased a lack of narrative coherence and the presence of tensions in their lives, while also emphasizing relational agency. The findings provide avenues for health, social, and justice practitioners who support and care for individuals living with HIV and a history of incarceration to think differently about transitions. By highlighting the importance of attending to the unique identities of individuals and relationships from a position of relational agency, the study advances our understanding of transitions. Recommendations for practice and policy include (a) fostering relational agency among practitioners; (b) challenging conventional views of transitions around correctional settings; (c) incorporating peer-based programming into support services; and (d) reconsidering health, justice, and social systems to better support communities disproportionately affected by high rates of incarceration and HIV.

## Introduction

Transitions into and out of correctional facilities are complex, and key gaps exist for those providing services in community and justice settings; specifically, the “coordination, oversight and monitoring of transitions in physical and mental health care” (Zinger, 2018, p. 98). In Canada, incarcerated populations face significant disparities in social determinants of health and experience more pronounced health inequities compared to the general population (Kouyoumdjian et al., 2016). These health disparities and knowledge gaps extend to people living with HIV (PLWH) undergoing care transitions (Moher et al., 2022; Pluznik et al., 2021; Woznica et al., 2021). Living with HIV exacerbates health and social inequities, presenting additional challenges for PLWH transitioning into and out of correctional facilities and communities (Iroh et al., 2015; Pluznik et al., 2021).

Transitioning into and staying within correctional facilities leads to increased antiretroviral therapy (ART) adherence and viral suppression rates among individuals living with HIV in Canada (Public Health Agency of Canada [PHAC], 2022; Subramanian et al., 2016). However, upon release from correctional centers, ART retention and viral suppression rates tend to decrease for PLWH, attributed to various individual and structural barriers (Iroh et al., 2015; Krsak et al., 2020; Moher et al., 2022; Pluznik et al., 2021; Taweh et al., 2021; Woznica et al., 2021). Although reinforcing ART adherence upon release is recognized as beneficial, many PLWH face significant barriers, including unstable social support networks, hindering their access to care despite acknowledging the importance of therapy adherence (Fuller et al., 2019; Pluznik et al., 2021; Woznica et al., 2021). Understanding transitions is of utmost importance as it facilitates improved interdisciplinary service coordination and practices to support incarcerated and previously incarcerated individuals (Zinger, 2018). Given the health and social inequities experienced by PLWH, transitions into and out of correctional facilities for this population represent a timely and critical area of prison research with important implications for policy and practice (Binswanger et al., 2012; Pluznik et al., 2021; Zinger, 2018).

In this article, we discuss a narrative inquiry into the experiences of transitions into and out of Alberta correctional facilities for PLWH. The inquiry was conducted from 2021 to 2022 in a Western Canadian city. Initially, we provide an overview of the incarceration landscape for PLWH in Canada and explore transitions concerning correctional facilities. A methodological discussion follows, outlining the inquiry process and the narrative threads that emerged through co-constructed narrative accounts with the participants living with HIV and a history of incarceration. Lastly, we offer insights on how a narrative conceptualization of transitions has the potential to shape care in new ways, particularly through the lens of relational agency. This approach views relationships as marked by trust and the capacity to develop agency between individuals, thereby shaping lives and facilitating collaborative services with PLWH and histories of incarceration.

## Background

### Living With HIV in Canada

At the end of 2020, Canada had an estimated 62,790 PLWH, with 1520 new HIV infections reported (PHAC, 2022). Key populations at higher risk of acquiring HIV include men who have sex with men, intravenous drug users, individuals in prisons, sex workers, and transgender people (World Health Organization [WHO], 2022). PLWH are diverse and often experience structural marginalization, which can include HIV-related stigma, gender-based power imbalances, or the criminalization of HIV transmission (Crable et al., 2021; Dauria et al., 2022; Montague et al., 2018; PHAC, 2015). Acquiring HIV leads to increased susceptibility to infections and certain cancers, and significant disparities in morbidity, mortality, stigmatization, and survival rates compared to the general population (Kouyoumdjian et al., 2020; PHAC, 2015; WHO, 2022).

Living with HIV has shifted, with treatment now making it a chronic, manageable disease offering more readily available and accessible treatment (for some populations) (Canadian AIDS Society, 2018; PHAC, 2015). “Treatment as Prevention” (TasP) or “Undetectable = Untransmissible” (U = U) highlights that HIV is untransmissible when individuals adhere to ART and maintain an undetectable viral load (Bavinton et al., 2018; Cohen et al., 2011; Rodger et al., 2016, 2019; Thompson et al., 2012). Canada endorses the 95-95-95 targets set out by The Joint United Nations Program on HIV/AIDS to improve the HIV care continuum: ensuring 95% know their status, 95% of those diagnosed are on ART, and 95% of those on ART achieve viral suppression (PHAC, 2022; UNAIDS, 2021).

### HIV and the Incarceration Landscape

Globally, rates of HIV among incarcerated individuals are six times higher than the general population (UNAIDS, 2021). These disparities originate from individual behaviors and conditions of living inside and outside of correctional facilities, specifically high-risk sexual behaviors (e.g., unprotected sex, multiple sexual partners, and sex while under the influence of substances) and injection drug use, as well as the lack of access to comprehensive needle exchange services in facilities (CATIE, 2015; Simonsen et al., 2015; Zinger, 2022). HIV-related stigma remains prevalent in Canadian and U.S. correctional facilities, leading to discrimination, abuse, and decreased willingness to seek health services for incarcerated and non-incarcerated PLWH (AVERT, 2022; Blue et al., 2022; Erickson et al., 2022; Muessig et al., 2016; Sprague et al., 2017; Wadams, 2022). HIV disproportionately affects incarcerated populations in Canada with a prevalence rate of ∼0.92% in correctional facilities compared to ∼0.17% in the general population (PHAC, 2022).

### Transitions Around Correctional Facilities

In 2020/2021, around 24% of adult custodial releases in Canada served 1 month or less (Statistics Canada, 2022). Remand centers (short-term housing facilities for those charged but not convicted) accounted for 67% of adults in custody in provincial/territorial facilities and represented 62% of total custodial admissions (Statistics Canada, 2022). The incarcerated population in Canada is diverse in ethnicity, race, and socioeconomic status (Dhaliwal & Hirst, 2016). Indigenous peoples in Canada are also grossly overrepresented, accounting for about 5% of the general Canadian population but making up approximately 31%–33% of corrections admissions nationally (Statistics Canada, 2022). PLWH are disproportionately represented among incarcerated individuals and pose specific challenges to the provision of care when undergoing transitions.

#### Transitions for People Living With HIV

Transitions into and out of correctional facilities are complex, involving social, health, and justice systems, with many individuals experiencing multiple health care transitions (Binswanger et al., 2012; Pluznik et al., 2021). For PLWH, these transitions are particularly challenging, with a higher risk of missed HIV care linkages upon release (Iroh et al., 2015; Loeliger et al., 2018; Pluznik et al., 2021). Contextual factors play a crucial role in shaping these experiences within Canadian correctional facilities and during the return to the community.

Transitioning into correctional centers in Canada and the United States presents an opportunity for diagnosing, engaging, and treating HIV, along with addressing other social and physical issues affecting HIV care outcomes (Dong et al., 2021; Iroh et al., 2015; Meyer et al., 2014; Milloy et al., 2014; Muessig et al., 2016; Sprague et al., 2017; Subramanian et al., 2016; Wood et al., 2009). Transitioning into facilities leads to increased ART adherence and viral suppression rates, partly due to reduced loss to follow-up, assured dosing schedules, and lower substance use (Milloy et al., 2014; PHAC, 2022; Subramanian et al., 2016). Alternatively, after release from correctional centers, PLWH experience declining ART retention and viral suppression rates due to individual and structural barriers, such as untreated SUDs, psychiatric disorders, homelessness, unemployment, housing instability, stigma, and comorbidities like HCV (Iroh et al., 2015; Kemnitz et al., 2017; Krsak et al., 2020; Moher et al., 2022; Montague et al., 2018; Pluznik et al., 2021; Springer et al., 2011; Swan, 2016; Taweh et al., 2021; Woznica et al., 2021).

Understanding transitions around correctional facilities is crucial for improving interdisciplinary service coordination and supporting incarcerated and previously incarcerated individuals (Zinger, 2018). HIV research in correctional settings tends to focus on increasing access to testing due to high rates of undiagnosed PLWH (Eastment et al., 2017; Iroh et al., 2015; Milloy et al., 2014). Research on transitions for PLWH primarily centers on transitions into the community, where viral suppression rates and ART adherence decrease (Woznica et al., 2021). Reinforcing ART adherence upon release is recognized as productive, but challenges, like unstable social support networks, hinder access to care (Fuller et al., 2019; Pluznik et al., 2021; Woznica et al., 2021). Therefore, addressing these gaps requires new interventions to improve and reconceptualize post-release HIV care (Crable et al., 2021; Iroh et al., 2015; Pluznik et al., 2021; Woznica et al., 2021), as well as an understanding of factors influencing post-release care, adherence, and viral suppression maintenance (Wohl et al., 2017).

This narrative inquiry alongside men living with HIV and histories of incarceration sought to understand their experiences of transitions and advance understandings of transition from a narrative perspective. The following questions guided the inquiry: what are the experiences of transitions into and out of Alberta correctional facilities for men living with HIV? What sense-making processes are involved for men living with HIV and histories of incarceration who experience inequities due to their social positioning? And what actions do they take to overcome these inequities?

## Methods

A one-and-a-half-year (spring of 2021 to fall of 2022) narrative inquiry was conducted in a Western Canadian city, exploring transitions into and out of correctional facilities by two men living with HIV. Narrative inquiry is a research methodology grounded in the traditions and work of Clandinin and Connelly (2000), Clandinin (2013), and Caine et al. (2022). Narrative inquiry is suitable for research alongside PLWH (dela Cruz et al., 2016; De Padua, 2015), addressing social justice and inequities while promoting personal growth (Clandinin & Caine, 2013; Clandinin & Rosiek, 2007). The inquiry was guided by an Indigenous Knowledge Keeper and Elder, who shared stories of experiences within and outside of correctional facilities and with Indigenous peoples living with and without HIV. Building connections and trusting relationships emerged as crucial throughout all phases of the study. Ethics approval was obtained through the University’s Research Ethics Board (Study ID: Pro00099605), along with operational approval from a community health center and local AIDS Service Organization (ASO). Written informed consent was obtained from participants before our conversations began. In this article, our theoretical orientation involves narrative ideas to conceptualize transitions and understand the health and social inequities faced by PLWH.

### Narrative Understanding of Experience

Narrative inquiry is rooted in a pragmatic framework, acknowledging that “what you see (and hear, feel, think, love, taste, despise, fear, etc.) is what you get” (Clandinin & Rosiek, 2007, p. 7). Turning toward narrative ideas involves more than the focus on an individual story and its associated characters, plot, or language used (Clandinin, 2013). It focuses on the complexity of a person’s life course, viewing experience as a narratively composed phenomenon (Clandinin, 2013). A number of ideas contribute toward this understanding in narrative inquiry (Clandinin & Connelly, 2000), such as Carr’s (1986b) work about the narrative structure and coherence of lives and Crites’ (1971) proposition that the formal quality of experience through time is inherently narrative. Yet, the theoretical orientation of this inquiry is primarily inspired by Dewey’s view of experience, emphasizing interaction and continuity in situations with a transactional or relational ontology (Caine et al., 2022; Clandinin, 2013).

Interaction involves simultaneous awareness of internal and external conditions of experience (Dewey, 1938). Continuity enacted in situations is the understanding that past experiences shape current experiences, while also laying the foundation for a plausible future (Clandinin & Rosiek, 2007). Narrative inquiry’s relational aspects stem from Dewey’s transactional and relational ontological commitment (1938); the regulative ideal of inquiry is to generate a new relation between a human and their environment, life, community, or world (Clandinin & Rosiek, 2007). These three aspects constitute narrative inquiry as a phenomenon and inform how individual experiences of transitions were theoretically constructed and approached in the study’s analysis.

### Recruitment

The inquiry included two participants; a sample this size is commonplace in narrative inquiry, because the design holds a longitudinal approach with ongoing engagement of participants within their social contexts (Clandinin, 2013; Clandinin & Connelly, 2000). Following institutional ethics review and approval, participants were recruited through purposive sampling, which is commonly found in narrative inquiry (Clandinin, 2013). Recruitment occurred through a local non-profit ASO. Both men were recruited one at a time; most of the energy of the project went into initially engaging and building relationships with each participant.

### Participants

Both men were living with HIV and a history of incarceration. Bruce was a heterosexual, Caucasian male, 65 years old, living with HIV for almost 19 years, and incarcerated three times across his lifespan. Kyle was around 46 years old, gay, and living with HIV since he was 18 years old and had spent over 18 years within provincial and federal facilities. Although the study did not specifically seek out Indigenous participants, Kyle was Indigenous, which is congruent with the disproportionate rates of Indigenous peoples incarcerated and living with HIV in Canada (PHAC, 2022; Statistics Canada, 2022). In line with the health inequities experienced by PLWH with current or past involvement in the penal system, both participants lived with/had lived with diverse comorbidities, such as substance use. The study excluded PLWH who identified as female or were diagnosed with a cognitive impairment.

### Data Collection and Fieldwork

During the fieldwork, I (first author) regularly met with Kyle and Bruce in person on multiple occasions. Bruce and I spoke of his family as we ran errands and spent time together out at his friend’s farm. Kyle and I mostly spoke together at a local fast-food place but would walk together to complete errands. Kyle and I met on 18 occasions while Bruce and I met 11 times. Meetings ranged from 30 minutes to over 4 hours. We co-composed field and interim research texts, engaging in conversations and unstructured dialogue documented via tape-recorded conversations and field notes, which allowed for the development of relationships and an unrestricted representation of their experiences. The longitudinal design of the study opened a space for collaboration, continued relationship-building, and the ability to revisit conversations. Tape-recorded conversations ranged from 20 minutes to 2 hours in length (dependent upon the participants availability and schedule) and were transcribed by the first author. These field texts, including autobiographical writing, journal entries, and personal artifacts, served as primary data sources and allowed their stories to be heard. I accompanied both participants wherever they took me, joining their social milieu and embedding their individual narratives within broader social, cultural, familial, political, and institutional contexts. We often revisited conversations from previous meetings, creating the opportunity to ask follow-up and clarification questions about their experiences of transition. Standing alongside Bruce as we waited for the bus together or walking with Kyle for him to have a smoke, the experiences and texts collected were subjective and open to interpretation, aligning with narrative inquiry’s emphasis on understanding experiences within their contexts (Clandinin & Caine, 2013; Clandinin & Connelly, 2000; Clandinin & Rosiek, 2007).

### Data Analysis

#### Field Texts to Narrative Accounts

Working from field texts to narrative accounts is an iterative process in narrative inquiry (Clandinin, 2013; Clandinin & Caine, 2013; Clandinin & Connelly, 2000). After collecting and sharing field texts, I would reshape my interpretation of their experiences based upon Kyle and Bruce’s feedback. From these field texts, narrative accounts were collaboratively written with Kyle and Bruce. The narrative accounts make sense of the diverse field texts and elicit additional experiences to be told, lived, retold, and relived (Clandinin, 2013; Clandinin & Caine, 2013; Clandinin & Connelly, 2000). Together, the participants and I co-composed the final narrative accounts and engaged in discussions to understand complexities and nuances while considering ideas of temporality, sociality, and place in their experiences (Clandinin & Caine, 2013). From the narrative accounts, narrative threads were created.

#### Narrative Threads

Participants’ narrative accounts were read and re-read to identify particular plotlines and echoes that extended across their stories, forming narrative threads (Clandinin, 2013). Narrative threads are not meant to be generalizations applicable to all PLWH in transition around correctional facilities but rather shared models of possibility (Bateson, 1990). Throughout this process, we were attentive to our relational responsibility to participants, respecting their stories and experiences (Clandinin & Connelly, 2000). Similarities, differences, and tensions in how the men made sense of and navigated their experiences of transitions were highlighted and discussed by the research team. The final analysis avoided reducing Bruce’s and Kyle’s experiences into pre-set categories, recognizing that transitions occur within the ongoing stream of their lives (Clandinin & Connelly, 2000). We approached the threads from a narrative understanding of experience.

## Findings

Engaging with Bruce and Kyle’s experiences throughout the inquiry offered insights into how they made sense of their experiences of transition around Alberta correctional facilities and living with HIV. Specifically, two narrative threads are discussed: (a) Bruce and Kyle’s experiences of going into and out of correctional facilities are part of their ordinary lives, but they encounter tensions—or a lack of narrative coherence—when confronted with dominant narratives and (b) the importance of relational agency in shaping their lives. These threads were approached from a narrative understanding of experience in narrative inquiry (Clandinin & Connelly, 2000), specifically the work of Carr (1986a, 1986b) and Crites (1971), as well as an emphasis on relationships and agency through the work of Edwards et al. (Edwards, 2005, 2011; Edwards & D’arcy, 2004; Edwards & Mackenzie, 2005). While both threads contribute to a narrative conceptualization of transitions, the emphasis of this work lies in how relational agency may shape a life.

### Tensions and Narrative Coherence

Stories are integral to how individuals make sense of their experiences, both as they are lived and as they are told (Carr, 1986b; Clandinin & Connelly, 2000; Connelly & Clandinin, 2006). Bruce and Kyle shared stories of transitioning into and out of correctional facilities, facing housing instability, HIV, and substance use, while also navigating street identities. Kyle does not do “dirt” (i.e., crime) that physically hurt others, yet he does do “dirt” to provide for himself. Bruce does not do “dirt,” as he believes in emanating good karma, but he faces housing instability, often lives outside, and faces predatory intrusions in his life (e.g., being pepper sprayed and robbed when sleeping outside). Tensions arose among the stories they lived and told, affecting their sense of narrative coherence (Carr, 1986a, 1986b).

Narrative coherence is crucial in understanding experiences from a narrative perspective (Carr, 1986a, 1986b). It involves purposefully making sense of an unfolding life that restructures, and it moves away from randomness and toward a sense of purpose and meaning. Achieving a sense of coherence is an ongoing pursuit, as individuals continuously make sense of their lives through improvisation and imagination (Carr, 1986b). For Kyle and Bruce, this pursuit of narrative coherence extended across their identities in both the street and “square” worlds.^1^

Kyle and Bruce shared stories of who they are and what they are about, and embedded in these stories were moments that lacked narrative coherence (Carr, 1986b). Their stories created tensions with dominant social, political, and familial narratives across street and “square” worlds. Kyle shared stories of being a sex worker:

To me, it doesn’t bug me but it’s part of my life. You know what I mean? It is the biggest page in my history book. It’s the longest chapter. The only chapter that I would probably ever write about, because the rest is just … fluff. You know what I mean? Like, I loved what I did. I made such good money, it wasn’t funny.

Dominant narratives impose expectations on intimacy, relationships, and bodies, influencing how they should be perceived and treated. For Kyle, living as a sex worker aligned with the street world but clashed with societal narratives around family and relationships. Meanwhile, Bruce’s choice to live outside—“one time, it was one week shy of an entire year in the river valley”—and found comfort in forested areas aligned with the street world’s sense of home but created tensions with dominant ideas around visibility and housing. Both Bruce and Kyle were constantly working toward reconciling their identities and lives with dominant narratives, seeking a sense of narrative coherence.

Achieving narrative coherence involves unifying three roles: the subject of a life-story, the principal teller of this story, and the audience to which it is told (Carr, 1986a). The story of one’s life is not only told to oneself but also to others, creating potential incongruences between these roles. Understanding how to unify these roles and navigate points of tension can shed light on how individuals strive for a sense of coherence as they transition in and out of correctional facilities.

Bruce and Kyle shared diverse stories ranging from selling thrills (i.e., sex, drugs, and excitement) to finding companionship and love. However, a lack of narrative coherence created disruptions and tensions in their lives. As they improvise and plan for future actions, we ask: how can their projected actions be shifted to achieve a sense of coherence? To gain a sense of coherence, it is important to acknowledge what gives birth to “the moment of decision within the story as a whole” (Crites, 1971, p. 303).

The moment of decision—our conscious present—is often characterized by a sense of uncertainty (Carr, 1986b; Crites, 1971). However, by engaging in relationships marked by trust, individuals like Kyle and Bruce can shift their sense-making in the present moment. Trust, arising from understanding their lives from a place of wholeness, contributes to a sense of coherence. Moving toward narrative coherence involves aligning oneself as a subject and storyteller with the audience, an ongoing endeavor. In this sense, trusting relationships with others (i.e., the audience) can lead to a consciousness that allows for new interpretations and actions in the world, fostering agency, hope, and increased capacity for change. These relationships may not always be familial but can be with others who offer support and new perspectives (Carr, 1986b).

### Relationships and Relational Agency

The second narrative thread that emerged in Kyle’s and Bruce’s accounts centered around their relationships with diverse individuals on changing landscapes. Notably, they emphasized two types of relationships: those with ASO outreach workers and community supervision officers. These relationships were a significant part of their experiences as they composed their lives and identities around correctional facilities and living with HIV. One particular relationship that stood out was with Cindy, an outreach worker at the ASO that both men attended.

Kyle: She has been a counselor at the network for over 20 years … Like, Cindy is my family.

This individual had created a relationship with Kyle that spanned two decades and is built on familial ideas. Bruce also shared a similar sentiment about Cindy and another outreach worker:

Bruce: They are my friends before my counselors. They are my friends that I ask questions when I need help.

The other relationship that resonated in their accounts were their probation officers:

Kyle: I had respect for her (Mary), and I never have respect for those kinds of people in that position, you know? In corrections of any sort, but her … I would definitely be friends with her on the street. She did try her hardest to work with me, and I appreciate that.

Arguably, Kyle and Mary disrupt the narratives of a community-supervised relationship where one holds a position of power and authority (i.e., ability to threaten one’s freedom or home) over the other. Bruce also shared a positive relationship with his last community supervision officer:

I had the best one ever … this last one I had 18 months probation. Her name was Jacky … She was great. She was absolutely the best.

As Bruce and Kyle storied these relationships, differences arose when compared to others that held positions of power, such as correctional or police officers. While formulating the narrative threads, questions arose: what was unique or empowering about these relationships with individuals who held positions of power/authority, such as Mary and Jacky, or were gatekeepers to resources and services, such as Cindy?

#### Relational Agency

Kyle’s and Bruce’s relationships with the outreach workers and their community supervision officers were marked by trust and the capacity to develop agency. This thread approaches the understanding of agency in the context of these relationships, and how one “gains” agency, through the idea of relational agency (Edwards, 2005, 2011; Edwards & D’arcy, 2004; Edwards & Mackenzie, 2005).

Relational agency was conceptualized in the field of teaching and learning but also draws on work among women facing social exclusion at an inner-city drop-in center (Edwards & D’arcy, 2004; Edwards & Mackenzie, 2005). Relational agency is “a capacity for working with others to strengthen purposeful responses to complex problems” (Edwards, 2011, p. 34); it occurs within a two-stage, dynamic process:

(i) working with others to expand the “object of activity” or task being worked on by recognizing the motives and the resources that others bring to bear as they, too, interpret it, and
(ii) aligning one’s own responses to the newly enhanced interpretations with the responses being made by the other professionals while acting on the expanded object (Edwards, 2011, p. 34).

Relational agency becomes essential in understanding how Bruce and Kyle gained the capacity to interpret and act upon their worlds differently. As a developed capacity, it allows individuals like Kyle and Bruce to interpret their experiences differently and take purposeful action. The “object of activity” refers to complex phenomenon or conditions of living, such as housing instability, adhering to ART, or following community supervision orders. “Professionals” in this understanding also include PLWH or histories of incarceration, that is, those we work with and alongside (Edwards, 2011, p. 34). The above-mentioned relationships, marked by hope and agency, were instrumental in shaping their lives amid the challenges of living with HIV and transitioning around correctional facilities. Relational agency changes someone’s trajectories of learning and positively impacts their ability to interpret a phenomenon, gather available resources, and then take purposeful action (Edwards & Mackenzie, 2005). This understanding is central in a narrative approach to transitions.

#### Dimensions of Relational Agency

Edwards and Mackenzie (2005) emphasize that our understanding of the world and our abilities within it are shaped by interactions with others and the materials they create. Thinking with Kyle’s and Bruce’s experiences, their transitions to the community often led to fragmented housing, social support, and economic conditions, similar to their experiences before entering correctional facilities. This draws our attention to what kind of support system we are transitioning people into if it remains fragmented or inadequate. Exploring the dimensions of relational agency can help make sense of their experiences.

##### Trust

Relational agency is dependent upon trust. Kyle and Bruce trusted other individuals, such as Cindy or Ashley (another outreach worker), to support them. When asked about this, Bruce responded:

Absolutely. Unequivocally … when I, umm, when I need to be grounded or muddled through things that are going on my life. I seek them. I talk to them.

Kyle and Bruce also trusted themselves above all else and saw themselves as their primary support during transitions into and out of correctional facilities. For example, Kyle shared, “Me, myself, and I. […] Plain and simple. I’ve never relied on others.” And Bruce stated, “I have always had a backup plan, whether it be to do it by myself or get it done by myself. […] I trust myself to do it.” They emphasized their self-reliance and the importance of having a backup plan. For service providers or agencies to connect with Kyle and Bruce, a relationship must include a sense of trust and belief in their agency. Demonstrating this belief can enhance their engagement with and transformation of their lives (Edwards & D’arcy, 2004). Trust is crucial in fostering relational agency and maintaining a relationship between individuals and those supporting them.

##### Actively Learning

Relational agency involves purposefully engaging and being open to relationships with others, recognizing available support, and acknowledging the purposes and direction of actions. In other words, one must be actively learning to engage in relational agency. In Kyle’s and Bruce’s experiences, Cindy plays a significant role in creating opportunities for engagement. Kyle actively reaches out to her, despite not engaging with others at her agency due to various social hierarchies (HIV-related stigma and street identities) he navigates: “I have never, ever talked with anybody there … I try not to be a snobbish person, but I can’t carry a conversation with them*.*” Bruce, on the other hand, is more actively involved with both the agency and Cindy, stopping by to bring fresh garden vegetables for their community kitchen or have a cup of coffee with others. Being purposefully active in each other’s worlds requires intentionality and the development of capacity to do so.

##### Agency as a Developed Capacity

Relational agency is an emergent capacity that involves learning and shifts in identity (Edwards & Mackenzie, 2005). Developing this capacity allows individuals to open themselves to other ways of knowing and being, especially in the context of lives and worlds different from their own. Throughout the inquiry, Kyle and Bruce were developing their capacity to engage in relational agency. Bruce shared:

You get to meet people on the street to exchange and trade ideas, because we aren’t so driven to pay rent or buy gas … your time is your time … we are not rushed … but it is different.

Engaging in and developing one’s capacity for relational agency can occur in various ways. Bruce actively engaged with people experiencing housing instability, embracing diverse ways of knowing and being alongside them. Social inclusion, a step toward relational agency, takes time to develop, and individuals’ trajectories of learning and capacity for relational agency evolve through active participation in social contexts in passive, peripheral, and active ways (Edwards & Mackenzie, 2005). Relational agency also involves reconfiguring social practices and results in gradual changes in a person’s identity. These changes may not follow linear paths but instead can be marked by periods of closeness and distance in relationships, reflecting the evolving capacity to engage in relational agency (Edwards & Mackenzie, 2005). The relationship between Kyle and Cindy, for instance, may have changed over time as their history suggests. Kyle would often try to support himself before turning toward their relationship:

I’ve never relied on others. Even when I get out, I don’t rely on these people. If there is something that Cindy can do for me, I will try, a million times, to do it—fall down and pick myself up and try again.

Relational agency is about working with others, but it’s important to note that Kyle does not solely *rely on these people* for support. However, more often than not, he includes Cindy and the support she offers when needed over other individuals and services. When Kyle turns to this relationship, he and Cindy collaborate on addressing housing instability and other challenges (i.e., objects of activity). In the context of the ASO and Cindy, it is essential to recognize that this inquiry only captured a short period of Bruce’s and Kyle’s life. Their relationships with Cindy and the organization have extended over two decades, and their capacity for relational agency has evolved over time and in different circumstances.

##### Spaces for Relational Agency

Individual learning trajectories can be influenced by the environment they are in (Edwards & Mackenzie, 2005). The inner-city drop-in center described in Edwards and others’ work served as a space that enabled relational agency. Over its 20-year existence, the center built strong relationships based on trust with community members, allowing workers to collaborate with them on various issues, including housing instability (Edwards, 2005). Much like the ASO and outreach workers in the inquiry, the center was accessible and flexible, fostering a sense of belonging and encouraging community members to develop their capacity for relational agency, eventually supporting newcomers to the center. This contrasts with formal institutions that community members also accessed, which may not foster the same affective and dialogic components of learning found in relationships (Edwards & Mackenzie, 2005). The drop-in center exemplifies how learning is not merely an individual endeavor. Purposeful spaces like this center can facilitate belonging and empower individuals to reshape how they interact with and perceive their worlds. Turning toward Bruce’s reflection on the ASO, he shared:

Bruce: It is more about a sense of belonging somewhere … they are helping me, but I try to help them in anyway for them … I just feel a sense of belonging there.

(First Author): Is there any other place that gives you that sense of belonging?

Bruce: No, not like that, no.

Relational agency thrives on the idea of having an open and welcoming space for relationships where individuals feel free to visit without needing a specific reason (Edwards & Mackenzie, 2005). Kyle and Bruce exemplify this understanding, knowing they can drop by the agency anytime without hesitation. The success of a center lies not only in providing physical resources but also in fostering relationships that enable individuals to understand and utilize those resources to expand their horizons (Edwards & Mackenzie, 2005). Creating an “open enough system” that encourages fluid relational agency is the ultimate goal of such spaces (Edwards & Mackenzie, 2005).

Relational agency in supportive spaces allows individuals to both succeed and learn from failures. Thinking with Kyle’s experiences, he perhaps understands he can attempt something on his own and fail because he has Cindy or the ASO to turn toward as support. Having a strong support network that engages in relational agency encourages risk-taking, exploration, and resilience in the face of challenges (Edwards & Mackenzie, 2005). In such environments, individuals feel safe trying new approaches, leading to personal growth and development (Edwards & Mackenzie, 2005).

##### Individualized Relations

Relational agency fosters a sense of togetherness and solidarity, shifting the focus from “I” to “we” in understanding and acting upon shared objectives (Edwards & Mackenzie, 2005). Building supportive, non-judgmental, and individualized relationships is crucial in this process, requiring service providers to be flexible, embrace uncertainty, and be open to building connections rather than adhering to rigid lesson plans or checklists (Edwards, 2005). In the relationships that Bruce and Kyle shared with their community supervision officers, it is arguable that Jacky and Mary did not approach Kyle’s and Bruce’s relationship as a checklist that warranted completion. Bruce shared an experience with Jacky:

Bruce: She was in my favor. In my court. I mean, I had an $18,0000 restitution order.

(First Author): What is that?

Bruce: I had to pay back … and her and her superior actually made that disappear. They knew the circumstances. I didn’t get any of that cash. I mean, it just went right through to whoever arranged all that stuff … So she knew that. I appreciate it, and it was the truth too. I didn’t see nothing from that except jail time—got that.

These ideas were also visible between Kyle and his probation officer, Mary:

In 2019, me and Mary were going to Gay Pride in Toronto, and I asked her (probation officer) if I needed permission due to curfew and stuff like that. She said, “No, but I will put a note into the police officers that you’re out of town. Not telling anyone where you’re going, cause ya know, it’s none of their business. Just go have fun, but don’t let me see you drunk or high.”

Mary’s understanding of Kyle’s need for privacy and her respectful approach toward authority figures like police officers demonstrate the importance of creating a space that accommodates flexibility. Individualizing care and respecting the priorities of Kyle and Bruce also extended to their relationships with outreach workers, specifically in the context of living with HIV. When asked the impacts of living with HIV on his life, Bruce responded:

It doesn’t affect my life … no, it did at first. Makes it that much harder … how do you disclose to a partner that you’re interested sexually? K? You know, that is a pretty crummy opening pickup line! (laughter).

While the realities of living with HIV-related stigma were emphasized, especially in the context of intimate relationships for both men, Bruce would often summarize his view toward living with HIV when compared to other illnesses:

I’d rather have this than anything else. It is nothing. Just one pill a day.

Bruce was consistently retained in HIV care prior to and throughout the study, often sharing his ongoing involvement:

I do pay attention to my health … if I do have vaccinations come up, like pneumonia or hepatitis shots or anything like that, I go ahead and take it. I keep my appointments when they’re made, and I pay attention and try to … make sure I am taking my meds. You know? Keep my numbers [CD4 count and viral load] where they are and I am happy.

However, even though his “numbers” are kept where he is happy, he continues to stay engaged with the ASO, seeking a personal connection with the agency and other PLWH—“It is more about a sense of belonging somewhere*.*” Bruce’s public connection to the agency contrasted with Kyle, who stayed away from creating connections with other PLWH:

I think it’s another reason why I don’t talk to people or clientele from there [the ASO]. If I see them in public, I don’t want them saying, “hi” to me. In public and if someone knows who they are, and “blah, blah, blah,” you know? “Oh, he goes to the clinic too.” It’s like, “no, no thank you.”

Although Kyle and Bruce were both consistently engaged in care throughout the inquiry, they exhibited differing needs related to living with HIV: Bruce sought connections with others, while Kyle preferred to keep his distance. Nevertheless, both men established long-lasting and meaningful relationships with the outreach workers from the ASO, who respected their ties to the organization and its community members. By acknowledging the entirety of Kyle’s and Bruce’s lives and providing a receptive and attentive audience for their stories (Carr, 1986a, 1986b), the outreach workers fostered relational agency and narrative coherence. While checklists used to guide transitional care services around correctional facilities may serve as useful starting points for newcomers in the field, they fall short of facilitating relationships that purposefully engage in relational agency.

##### Reciprocity

Reciprocity is a key aspect of relational agency (Edwards, 2005). It counters dependency by fostering resilience and growing independence in individuals, enabling them to support others while evolving their own sense of self (Edwards, 2005). In the drop-in center, reciprocity was evident as individuals became helpers, drawing upon available resources and connecting others to them. Bruce also shared his network of supports with others experiencing housing instability. This reciprocal approach builds resilience in others and shapes individuals’ identities within relationships (Edwards & Mackenzie, 2005). Trust and changes in a sense of self are the primary gifts exchanged in these reciprocal relationships. For example, Bruce’s trust in Cindy and others at the ASO deepened over time—“There is total trust, more and more so all the time”—while Kyle considered Cindy as family, reflecting the reciprocity of trust and emotional connection through their use of humor with one another.

Interacting with others creates an opportunity to open one’s life to an audience, shifting one’s sense of self and the “moment of decision” within a story (Carr, 1986a, 1986b; Crites, 1971). Relational knowing and an individual’s capacity to engage and interact in and with their world(s) is enlarged when relationships are framed by the dimensions of relational agency. Emphasizing togetherness, flexibility, trust, and reciprocity, relational agency fosters an open and responsive environment where individuals can develop a deeper understanding of themselves and others, leading to transformative experiences and enhanced support networks. These understandings help us understand the stories that both men told, as well as the relationships that shaped their lives. Both narrative threads add to a further understanding of a narrative conceptualization of transitions that may guide future work.

## Discussion

The objective of narrative inquiry is to enhance human experience by utilizing knowledge in a meaningful way (Clandinin & Rosiek, 2007). Rather than seeking generalizations, narrative inquiry aims to explore new perspectives on issues, generating new insights (Clandinin, 2013; Clandinin & Connelly, 2000). In response to the insufficient improvements in community transitions for Indigenous peoples from correctional facilities, Zinger (2017) remarked, “It is clear that more of the same will not produce better or different results” (p. 51). We reflect on this statement and how the findings from the inquiry, specifically the dimensions of relational agency, can provide a starting place to rethink and imagine otherwise transitions into and out of correctional facilities for PLWH. Four recommendations were developed: engaging in relational agency between practitioners, disrupting the traditionally “successful” transitions around correctional facilities, integrating peer-based programming, and rethinking systems and supporting communities.

### Relational Agency Between Practitioners

Practitioners, such as outreach workers, social workers, community supervision officers, and nurses, operate across various disciplines and geographical areas, engaging with numerous social, health, and justice organizations. This interdisciplinary work is inherently complex and demanding. Practitioners involved in care can purposefully engage in relationships with other professionals that attend to the dimensions of relational agency (Edwards, 2005). Specifically, community supervision officers contribute diverse perspectives and specialized knowledge, which are crucial for understanding and supporting individuals during their transitions around correctional facilities.

The role of community supervision officers in supporting individuals transitioning from correctional facilities is critical, particularly in building personalized relationships (Chamberlain et al., 2018; Green et al., 2013; Kennealy et al., 2012). Trusting relationships with these officers are key to reducing rearrest and recidivism among people with incarceration histories (Chamberlain et al., 2018; Kennealy et al., 2012). For instance, Kyle noted his general distrust of authority figures, yet, like Mary, community supervision officers can challenge conventional views on authority within relationships. Mary and Jacky respected Bruce and Kyle and facilitated an increased capacity to practice agency in their lives. This facilitation of agency not only benefits the individuals directly involved but also informs how practitioners interact across different disciplines.

Relational agency among practitioners encompasses several key aspects: focusing on the individual (i.e., client or community member) within their broader living conditions, clarifying work objectives, openness to alternative approaches, adopting a pedagogic stance, responsiveness, engaging in rule-bending and risk-taking, developing knowledge-sharing processes, and learning from practice (Edwards, 2011). Specifically, when professionals collaborate on client learning trajectories, their collective expertise should be integrated, emphasizing the coordination of support (Edwards, 2005). Practitioners must view each other as resources and synchronize their efforts effectively (Edwards, 2005). This approach forefronts relational agency and aligns with calls for an integrated strategy that bridges correctional and community healthcare (Hickey et al., 2017; Woznica et al., 2021), emphasizing the need to extend relationships beyond correctional facilities in order to provide inclusive services for PLWH and incarceration histories.

### Disrupting a “Successful” Transition

Traditionally, “successful” transitions between correctional facilities and communities for PLWH have been gauged by the HIV care continuum’s evaluative criteria (Baillargeon et al., 2010; Dong et al., 2021; Gardner et al., 2011; Kouyoumdjian et al., 2020; Meyer et al., 2014; Subramanian et al., 2016), which often continues linear trajectories of care within segregating metaphors of inside versus outside facilities (Wadams, 2021). Within correctional settings, disciplinary efforts have predominantly focused on implementing and evaluating interventions to enhance HIV testing (Eastment et al., 2017; Iroh et al., 2015; Milloy et al., 2014). Research on community transitions has primarily aimed to meet HIV care continuum targets, such as viral suppression, by addressing individual and structural barriers to continuous care (Moher et al., 2022; Woznica et al., 2021). Consequently, transitional care for PLWH often revolves around these targets: Does the patient have a 3-day supply of ART medication? Is there a nearby pharmacy they can use to pick up their medications? Do they have a referral to an outside HIV care physician, or are they booked to see the physician within the facility? Do they have a home to stay at, or if not, do they have the number of a shelter to call? While these discharge summaries and health assessment forms provide a structural basis for clinical practice, this cannot be our understanding of people living in transition. Bruce’s and Kyle’s experiences disrupt these practices.

Kyle and Bruce were men living with HIV, and while the literature highlighted the significance of living with HIV in relation to policies guiding transitions (Iroh et al., 2015; Pluznik et al., 2021; Woznica et al., 2021), their experiences disrupted this emphasis. Bruce and Kyle were more than their diagnosis, and while living with HIV impacted their lives, it was not to the extent that their lives revolved around this illness. Discussing tasks like attending appointments or taking medications, they expressed these activities around correctional facilities as inconvenient rather than central to their existence.^2^ Yet, their stories also reveal significant barriers such as HIV-related stigma, housing instability, and a lack of trusting relationships, reflecting broader structural and individual challenges faced by PLWH (Fuller et al., 2019; Kemnitz et al., 2017; Lim et al., 2015; Pluznik et al., 2021; Rozanova et al., 2015; Woznica et al., 2021). While the literature focuses on navigating these barriers in the context of providing and retaining PLWH in HIV care trajectories, it begs the question: if PLWH were to create their own transition checklists and discharge summaries, would the emphasis on HIV care persist? What additional concerns might they highlight?

If health, justice, and social services aim to provide comprehensive support across various settings, then the stories of individuals like Kyle and Bruce must inform these efforts. While recognizing the challenges practitioners face in building trusting and flexible relationships within constrained resources and time limits, embracing relational agency is essential. Starting with the lived experiences of those in transition can disrupt current practices and offer a new lens through which to consider health, social, and justice policies. We recommend that practitioners involved in correctional service policy development and community integration processes incorporate cross-disciplinary priorities that include the stories of community organizations and affected individuals to thoughtfully reshape these policies and practices.

### Integrating Peer-Based Programming

An individual’s trajectory of care or social inclusion is dynamic, continually presenting new possibilities, challenges, and opportunities for both the individuals and those supporting them (Edwards, 2005). Bruce and Kyle’s transition experiences extended beyond the confines of penal facilities; therefore, relationships, interventions, and programs designed to support them should also transcend these boundaries. Consistent with this perspective is the principle that effective linkage to care post-release should initiate at the time of admission to facilities (Krsak et al., 2020). Fernando et al. (2022) recognize the importance of establishing supports before and during incarceration, focusing particularly on building relational networks through peer-based programming that persists throughout incarceration and following release.

Since the 1980s, peer-based programming, or peer work, has been integrated with HIV care services to achieve HIV care continuum targets through various means such as health education, outreach, navigation services, and research (Tobias et al., 2010). At the core of peer work is the establishment of strong, empathetic relationships. Building on this, relational agency emphasizes the significance of sustained relationships and enhancing the ability to access pre-incarceration resources and supports. Peer-based programs that extend within and across correctional facilities and foster relational agency among people with similar experiences can play a crucial role in meeting HIV care continuum targets (Dauria et al., 2022; Fernando et al., 2022; Koester et al., 2014; Moher et al., 2022; Myers et al., 2017; Ostermann et al., 2021; Woznica et al., 2021) and in developing individual agency. Organizations and individuals facilitating peer-based programs should ensure they provide a diverse, flexible mix of formal and informal services both inside and outside of facilities that respond to locally identified priorities.

While existing research highlights the impact of relationships on the care trajectories of PLWH with histories of incarceration (Rozanova et al., 2015; Woznica et al., 2021), family or close social supports often are not available to support previously incarcerated PLWH upon release (Rozanova et al., 2015). Reflecting on Kyle’s experiences, it is crucial for individuals leading peer programs and developing relationships to share similar “worlds” (e.g., street and square) with participants. While we recognize that practices within and outside correctional facilities differ significantly due to traditional focuses on social control (Holmes et al., 2007), we believe that adopting a narrative approach that foregrounds relational agency offers a fresh perspective that can enhance peer-based programming.

### Rethinking Systems and Supporting Communities

Throughout the inquiry, the experiences and narratives of Kyle and Bruce underscored the importance of establishing supports that persist post-release as individuals reintegrate into their communities. By adopting a relational agency approach, we can highlight alternative care trajectories around correctional facilities, diverging from current practices. A thorough understanding of individuals’ lives before and after incarceration necessitates a careful consideration of their evolving identities. Outreach workers in our study demonstrated this understanding by maintaining connections with participants during their incarceration, upon release, and throughout their reintegration into the community. These workers were keenly aware of the stigma previously incarcerated PLWH likely face when accessing health services (Haley et al., 2014; Swan, 2016), highlighting the need for supportive systems and relationships that extend beyond the walls of correctional facilities. Our modest recommendation is to encourage practitioners and care providers to facilitate regular connections and conversations with not only patients and clients but also the community organizations and individuals that provide care outside of correctional facilities. Whether this is a local ASO like the one in the inquiry, or a community health center or other non-profit that connects with community members with histories of incarceration. We also encourage the establishment of clear communication lines between correctional services and outside community organizations, potentially through the use of regular patient/caseload meetings or transition planning. Finally, we recommend starting with the patient or community member, asking them who their preferred service providers and individuals are in the community or correctional facility, and connecting with these individuals as relationships should follow a life throughout their incarceration and release.

The conditions of living for someone upon arrest are the same conditions upon release; social change occurs inside and outside of facilities. Both men were deeply affected by their living conditions within communities characterized by diverse social groups. Reflecting on the men’s experiences, the racial disparities facing specific communities in Canada are highlighted; Indigenous peoples face inequities in the social determinants of health (Kolahdooz et al., 2015; Kouyoumdjian et al., 2016) and disproportionate rates of HIV and incarceration (PHAC, 2022; Statistics Canada, 2022). This underscores the urgent need for systemic reform and enhanced support for Indigenous communities and peoples.

Without opportunities for growth within the communities that individuals leave and return to, existing health and social inequities will persist. Health, social, and justice systems must recognize the importance of long-term engagement with communities. Specifically, social initiatives should focus on building relationships, sharing decision-making, fostering self-determination, and increasing agency through solidarity and community mobilization in communities disproportionately affected by HIV (DiCarlo et al., 2022; UNAIDS, 2007). By adopting relational agency, we can start addressing these challenges. However, these efforts must intentionally involve communities disproportionately affected by high rates of HIV and incarceration. If systems aim to reduce recidivism (Government of Canada, 2023), meet the 95-95-95 targets guiding HIV policy (PHAC, 2022), or rethink transitions from the perspective of individual lives, they should begin by developing programs that leverage and are based upon the dimensions of relational agency. These programs should engage stakeholders and community organizations in developing transition protocols and policies and explore collaborative opportunities among correctional services, community-based organizations, and academia through potential grant funding and other avenues to augment service provision. The stories and experiences of Bruce and Kyle highlight that relationships are built one at a time and offer valuable insights into conceptualizing policies and frameworks that address the needs of PLWH with a history of incarceration.

### Limitations

This inquiry was conducted in a Western Canadian city with two men living with HIV and histories of incarceration. The sample size is small, but appropriate for narrative inquiry, which utilizes a longitudinal design focused on relational knowing of participants in their social contexts (Clandinin, 2013; Clandinin & Connelly, 2000). Small sample sizes are justifiable in qualitative research when the intent is to deliver an in-depth and novel understanding of phenomena (Boddy, 2016). The narrative threads presented in this study are not meant to be universally applicable to all PLWH living in transition; instead, they offer a starting point to explore alternative perspectives on transitions around correctional facilities from a place of “otherwise” (Greene, 1995). However, their stories provide valuable insights for rethinking and approaching trajectories of care around correctional facilities. Reflecting on their experiences, it becomes evident that their lives and identities were continuously evolving and “in the making” (Clandinin & Connelly, 2000).

Acknowledging the complexities involved in proposing major systemic changes based on the experiences of Kyle and Bruce, we aim to identify potential areas for improvement within the criminal justice and health systems. We advocate for feasible, incremental changes that acknowledge the entrenched structures and complex nature of these systems. Meaningful and lasting changes necessitate an understanding of the intersecting layers of vulnerability, strength, and marginalization affecting not only PLWH but also those with histories of incarceration (Moher et al., 2022). Furthermore, it is crucial for services and disciplines to foster meaningful and transparent communication that transcends the physical boundaries of correctional facilities and bridges diverse healthcare organizations, non-profits, and social services (Hickey et al., 2017; Woznica et al., 2021). We recognize the scarcity of resources that complicates efforts to implement these changes. Although existing strains on our work and caseloads limit the time we can spend with patients or clients, we believe that developing trusting relationships that embody relational agency falls within our scope of practice. By strategically allocating resources to practitioners and services involved in transitional care—where not only gaps in services exist but also opportunities to engage and retain those in care (Moher et al., 2022; Woznica et al., 2021)—meaningful change can begin to take root, one relationship at a time.

The inquiry focused on the experiences of Bruce and Kyle, but future work would benefit from exploring the perspectives of service providers, within and outside of correctional facilities, on their experiences of transitions. We also acknowledge that relational agency takes different forms (Edwards & Mackenzie, 2005) in diverse relationships. The participants’ experiences may also differ from other Indigenous peoples or racial minorities within or outside of correctional settings, as well as along gender lines. All these potential avenues are areas of future growth and inquiry warranting further exploration.

## Conclusion

In the context of men living with HIV, histories of incarceration, and a life course that is often chaotic, accessing and remaining engaged in health and social care services pose considerable challenges. Understanding and improving care trajectories around correctional facilities in Canada necessitates fostering relationships with various service providers both within and outside the facilities. Recognizing that the lives of Bruce and Kyle existed before and will continue after incarceration underscores the need for continuous support through their transitional phases. The men’s experiences highlight the tensions and potential disconnections in individuals’ lives, which, while challenging, also open opportunities for transformative shifts in self-perception, intertwining the “I” and “we” in meaningful ways. Engaging with the dimensions of relational agency illuminates the importance of intentional relationship-building and understanding diverse life trajectories. Community supervision officers and outreach workers, by practicing relational agency in their interactions with Bruce and Kyle, fostered a sense of narrative coherence for the men. Adopting a narrative approach to transitions could profoundly transform care trajectories and address health inequities. To cultivate a more integrated and empathetic approach to transitional care that reflects the complex realities of life before and after incarceration, we advocate for a narrative approach that enhances relational agency among practitioners, challenges conventional views of transitions around correctional settings, incorporates peer-based programming into support services, and reconsiders health, justice, and social systems to better support communities disproportionately affected by high rates of incarceration and HIV. This inquiry encourages further discussion and creates opportunities for practitioners, academics, and policymakers to reevaluate their methods of forming and maintaining relationships with PLWH and histories of incarceration during transitions in Canada.

## Notes

1. Prison subcultures and their evolving languages have been well-documented (Bell et al., 2022; Gibbons & Garrity, 1962; Irwin & Cressey, 1962). For instance, the term “Square John” is used to describe individuals with law-abiding tendencies or situational offenders who are not deeply involved in criminal subcultures (Irwin & Cressey, 1962; Wheeler & Cline, 2020). Over the inquiry, Kyle frequently utilized the term “Square” to denote those who are not primarily engaged in street life.

2. Although Kyle and Bruce shared no difficulties accessing HIV care within facilities, we recognize that access varies widely across different institutions and countries due to factors like funding structures and incarceration length (Blue et al., 2022).
